# Neonatal-onset cblC-type methylmalonic acidemia combined with homocysteinemia: case report

**DOI:** 10.3389/fped.2026.1878193

**Published:** 2026-07-29

**Authors:** Guoqiang Zhang, Liming Zhang, Hong Liu

**Affiliations:** 1 Department of Neonatology, Weifang People's Hospital, Weifang, Shandong, China; 2 Department of Pediatrics, Weifang People's Hospital, Weifang, Shandong, China

**Keywords:** case report, homocysteinemia, methylmalonic acidemia, *MMACHC* gene, neonate

## Abstract

**Objective:**

To investigate the clinical diagnostic features, screening and definitive diagnostic methods of neonatal-onset cblC type methylmalonic acidemia (MMA) combined with homocysteinemia, so as to provide a reference for early clinical identification and diagnosis of this disease.

**Methods:**

The clinical data of 1 neonate with neonatal-onset cblC type MMA combined with homocysteinemia admitted to our hospital were retrospectively analyzed. Routine laboratory tests, blood tandem mass spectrometry, serum homocysteine test, urinary organic acid analysis, cranial MRI and genetic testing were performed on the neonate. Combined with relevant literature, the clinical features, diagnostic strategies, treatment and prognosis of the disease were reviewed and summarized.

**Results:**

The patient was a 20-day-old male neonate, presenting with persistent feeding difficulty and weak response after birth as the main manifestations, accompanied by hypotonia and diminished primitive reflexes, without typical critical signs. Laboratory examinations showed significantly elevated serum homocysteine; blood tandem mass spectrometry revealed increased propionylcarnitine, elevated C3/C2 ratio and decreased methionine; urinary organic acids showed increased methylmalonic acid and methylcitric acid. Cranial MRI showed hyperintensity in the bilateral globus pallidus on T1WI. Genetic testing identified two heterozygous pathogenic variants, c.567dupT and c.609G>A, in the *MMACHC* gene of the neonate, inherited from the mother and father respectively, conforming to the autosomal recessive inheritance pattern. The patient was finally diagnosed with cblC type MMA combined with homocysteinemia.

**Conclusion:**

Neonatal-onset cblC type MMA combined with homocysteinemia has insidious and non-specific clinical manifestations, which are easily confused with other neonatal diseases, leading to missed diagnosis and misdiagnosis. For neonates with unexplained feeding difficulty, poor mental response and abnormal muscle tone, timely metabolic screening including blood tandem mass spectrometry, urinary organic acid analysis and serum homocysteine test should be conducted. Genetic testing is the gold standard for the definitive diagnosis and classification of this disease, and can also provide a basis for genetic counseling and prenatal diagnosis. The implementation of neonatal screening for inherited metabolic diseases and the establishment of a multidisciplinary collaborative diagnosis and treatment model are the keys to achieving early diagnosis and treatment of this disease and improving the prognosis of patients.

## 1 Introduction

Methylmalonic acidemia (MMA) is the most common disease among congenital organic acid metabolism disorders, inherited in an autosomal recessive manner. According to whether it is complicated by elevated homocysteine, MMA can be divided into isolated MMA and combined MMA. Among them, cblC type is the most common subtype of combined MMA, caused by *MMACHC* gene mutation leading to cobalamin metabolism disorder. The prevalence of MMA varies greatly worldwide. It is reported that the prevalence of MMA is about 1:22,727 in Italy (1), 1:158,730 in the United States (2) and 1:120,000 in Japan (3). The overall incidence of methylmalonic acidemia identified via nationwide Chinese newborn screening programs is approximately 1 in 29,000 live births, with striking regional disparities. Northern provinces exhibit a much higher prevalence ranging from 1 in 6,000 to 1 in 30,000, while the incidence remains relatively low across southern regions (4). Neonatal-onset cblC type MMA is often critical, with non-specific clinical manifestations, difficult early diagnosis, and high mortality and morbidity. Herein, we report a patient with neonatal-onset disease predominantly presenting with feeding difficulties and non-specific neurological abnormalities. Distinctively, this infant exhibited an early disease onset without typical critical manifestations such as convulsions, severe metabolic acidosis, or hyperammonemia, which have been frequently documented in previous case series. Nevertheless, such atypical clinical presentations are not rare in neonates and are readily misdiagnosed as neonatal sepsis (early- or late-onset), hypoxic-ischemic encephalopathy, inappropriate feeding, congenital myopathy, Prader–Willi syndrome, or Angelman syndrome.

## 2 Subjects and methods

### 2.1 Subjects

A 20-day-old male neonate was admitted to the Neonatal Intensive Care Unit of our hospital due to “feeding difficulty and weak response for 20 days”. The neonate was the second child of the fourth pregnancy, with a gestational age of 38 weeks, delivered vaginally due to the mother's “premature rupture of membranes, gestational diabetes mellitus, and cervical cerclage during pregnancy”. The birth weight was 2,870 g, and all Apgar scores were 10 after birth. The neonate was fed with mixed feeding on the day of birth, and presented with weak sucking, weak crying, unsatisfactory weight gain, drowsiness, weak response and hypotonia immediately after birth. The neonate was treated with blue light for “neonatal hyperbilirubinemia” after birth, but feeding difficulty and weak response persisted. Family history: the parents were non-consanguineous and healthy. The mother had gestational diabetes mellitus and 2 unintended pregnancy abortions. The first child of the mother was a boy, who was found to have hydrocephalus at 1 month after birth and died at the age of 9 years (unknown specific cause). Informed consent was signed by the parents for the diagnosis and treatment of the neonate, and the study was approved by the hospital ethics committee.

### 2.2 Methods

1. Routine laboratory tests: including blood routine, blood gas analysis, liver and kidney function, electrolytes, blood glucose, blood ammonia, lactic acid, myocardial enzymes, etc.
2. Blood tandem mass spectrometry analysis: Tandem mass spectrometry analysis of dried blood spots was performed using a Waters TQD triple quadrupole mass spectrometer (Waters Corporation, USA) with the NeoBase™ MSMS Kit (PerkinElmer). Heel peripheral blood was collected to make dried blood filter paper, and high-performance liquid chromatography-tandem mass spectrometry was used to analyze blood amino acids and acylcarnitine profiles. The levels of propionylcarnitine (C3), acetylcarnitine (C2), methionine (Met) and C3/C2 ratio were mainly observed.
3. Serum homocysteine test: Peripheral venous blood was collected, and the circulating enzyme method (two-point rate method) was used to measure the total plasma homocysteine level.
4. Urinary organic acid analysis: Urine organic acid profiling was performed using a gas chromatography-mass spectrometry (GC-MS) system. Fresh urine was collected, the levels of methylmalonic acid, 3-hydroxypropionic acid and methylcitric acid were mainly observed.
5. Neuroimaging examination: Cranial magnetic resonance imaging (MRI) was performed to evaluate brain structural development.
6. Genetic testing: Whole-exome sequencing was conducted on the NovaSeq 6,000 sequencing platform (Illumina, USA). All raw data were aligned against the human reference genome UCSC hg19 with the Burrows-Wheeler Aligner. The Genome Analysis Toolkit (GATK) was utilized to identify insertion and deletion variants, followed by genetic variant annotation via ANNOVAR. Peripheral venous blood of the neonate and his parents was collected to extract genomic DNA. Whole-exome sequencing (trio family analysis of the proband and his parents) was performed, and the full-length mitochondrial genomic DNA of the neonate was also sequenced. Point mutations and small fragment insertion-deletion mutations in the nuclear genome and mitochondrial genome were analyzed based on next-generation sequencing data. Suspected pathogenic mutations were verified by Sanger sequencing, and family co-segregation analysis was performed.

## 3 Results

### 3.1 General condition and physical examination of the neonate

Admission condition: admitted due to feeding difficulty and poor response for more than ten days. Physical examination: body temperature 36.9 ℃, pulse 120 beats/min, respiration 40 breaths/min, weight 2.8 kg. Clear consciousness, poor mental response, slight yellowish skin, no skull deformity, flat and soft anterior fontanelle (about 1.5 cm × 1.5 cm), soft neck, clear bilateral lung breath sounds without rales. Heart rate 120 beats/min, strong heart sound, regular rhythm, no murmur. Flat and soft abdomen, no hepatosplenomegaly. Hypotonia of limbs, weak primitive reflexes (embrace, sucking, grasp, rooting reflexes). No simian crease. The neonate was admitted for “neonatal hyperbilirubinemia” previously.

### 3.2 Laboratory test results

1. Blood routine, blood gas, electrolytes, liver function, kidney function, myocardial enzymes, blood ammonia and lactic acid: within normal range.
2. Serum homocysteine: 203.94 μmol/L (reference range: 0–17 μmol/L).
3. Blood tandem mass spectrometry analysis: elevated propionylcarnitine (C3): 7.84 μmol/L (reference range: 0.30–4.50 μmol/L); normal acetylcarnitine (C2): 13.07 μmol/L (reference range: 5–65 μmol/L); decreased methionine (Met): 4.18 μmol/L (reference range: 7.00–50.00 μmol/L); elevated C3/C2 ratio: 0.6 (reference range: 0.03–0.18).
4. Urinary organic acid analysis: elevated methylmalonic acid: 186.57 μmol/L (reference range: 0–4.00 μmol/L); 3-hydroxypropionic acid: 2.9 μmol/L (reference range: 0–4.00 μmol/L); elevated methylcitric acid: 8.66 μmol/L (reference range: 0–1.5 μmol/L).

### 3.3 Neuroimaging examination result

**Figure 1:**
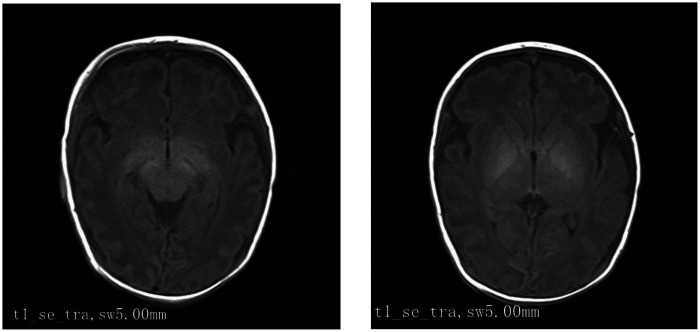
Cranial MRI revealed hyperintensity of bilateral globus pallidus on T1WI.

Cranial MRI showed hyperintensity in the bilateral globus pallidus on T1WI (Figure 1).

### 3.4 Genetic testing result

Whole-exome sequencing (family analysis) showed that the neonate had two heterozygous pathogenic variants in the *MMACHC* gene (Table 1):

1. NM_015506.3: c.567dupT (p.Ile190Tyrfs*13), located in exon 4, a frameshift mutation leading to premature termination of protein translation. This variant was inherited from the mother and was a pathogenic variant.
2. NM_015506.3: c.609G>A (p.Trp203*), located in exon 4, a nonsense mutation leading to premature termination of protein translation. This variant was inherited from the father and was a pathogenic variant.

**Table 1:** Nuclear genomic point mutations and small-fragment insertion-deletion ariants (SNVs).

Gene	Chromosomal location	Variant information	Zygosity	Disease name	Inheritance mode	Variant origin	Variant classification
*MMACHC*	chr1: 45974605	NM_015506.3: c.567dup (p.Ile190Tyrfs*13)	Heterozygous	Methylmalonic aciduria and homocystinuria, cblC type [MIM:277400]	AR	Maternal	Pathogenic variant
*MMACHC*	chr1: 45974647	NM_015506.3: c.609G>A (p.Trp203*)	Heterozygous	Methylmalonic aciduria and homocystinuria, cblC type [MIM:277400]	AR	Paternal	Pathogenic variant

Family Sanger sequencing verification confirmed that the above variants were derived from the parents respectively (Table 2), conforming to the autosomal recessive inheritance pattern. Combined with clinical manifestations, biochemical metabolic abnormalities and genetic testing results, the patient was finally diagnosed with “cblC type methylmalonic acidemia combined with homocysteinemia (MAHCC)”.

**Table 2:** Sanger sequencing chromatogram.

Sanger sequencing validation results					
Validation of variant locus information				Relationship	Validation results
MMACHC chr1:45974605 Exon:4/4 NM_015506.3: c.567dup (p.Ile190Tyrfs*13)				proband	heterozygous duplication
				father	wild-type
				mother	heterozygous duplication
MMACHC chr1:45974647 Exon:4/4 NM_015506.3: c.609G>A (p.Trp203*)				proband	heterozygous
				father	heterozygous
				mother	wild-type
Chromatogram verification results					
Gene	MMACHC	Genomic coordinate	chr1:45974605	Variant information	c.567dup (p.Ile190Tyrfs*13)
Proband, Forward Sequencing Heterozygous Duplication		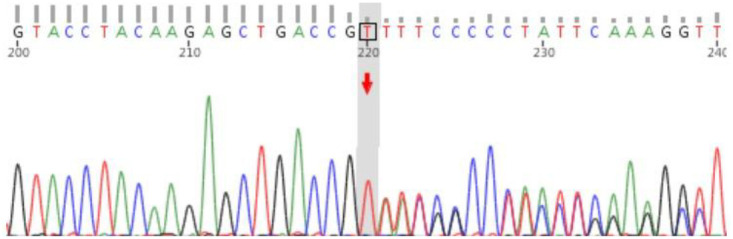			
Proband, Reverse Sequencing Heterozygous Duplication		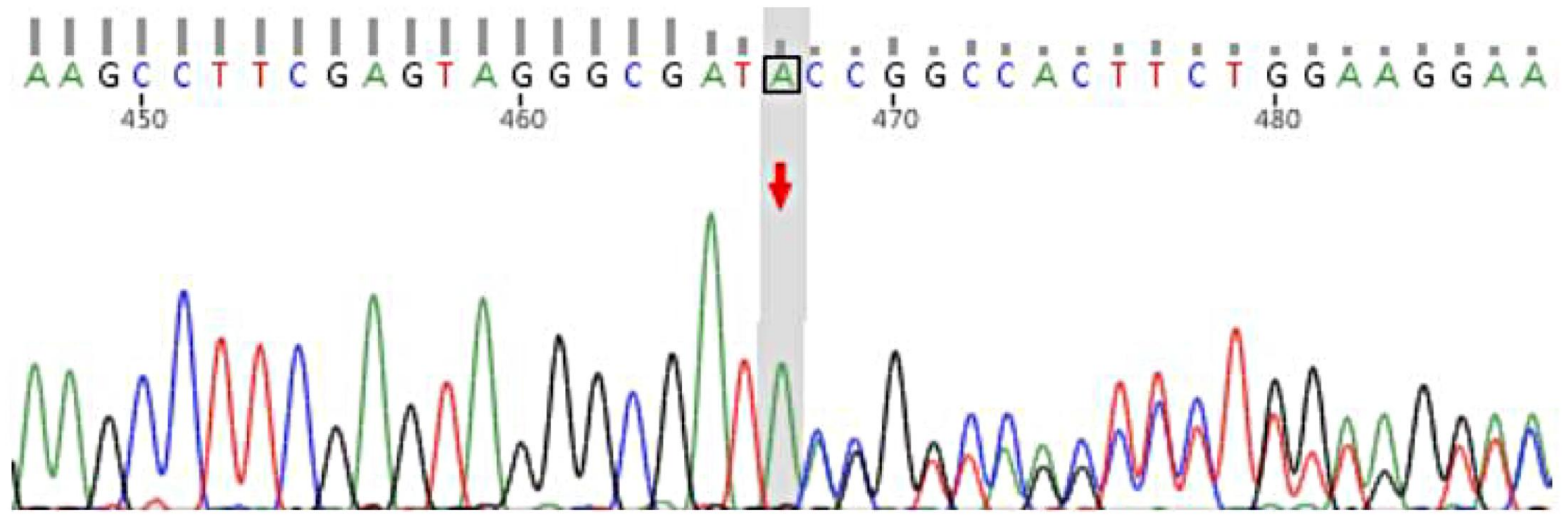			
Father, Forward Sequencing Wild-type		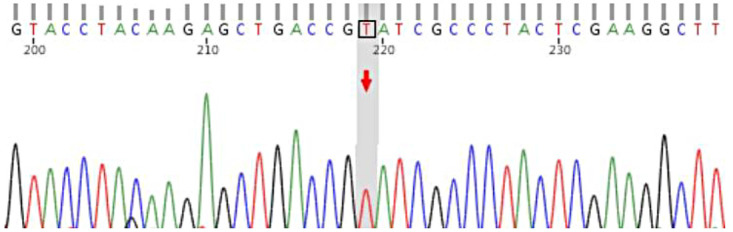			
Mother, Forward Sequencing Heterozygous Duplication		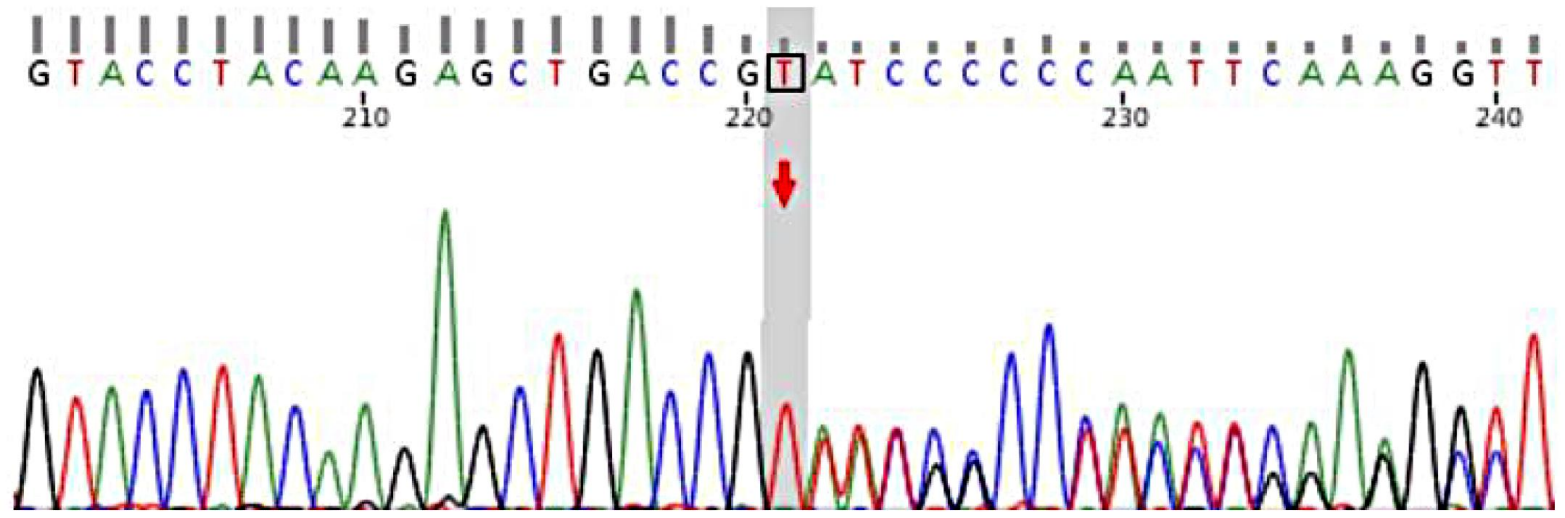			
Chromatogram verification results					
Gene	MMACHC	Genomic coordinate	chr1:45974647	Variant information	c.609G>A (p.Trp203*)
Proband, Reverse Sequencing Heterozygous		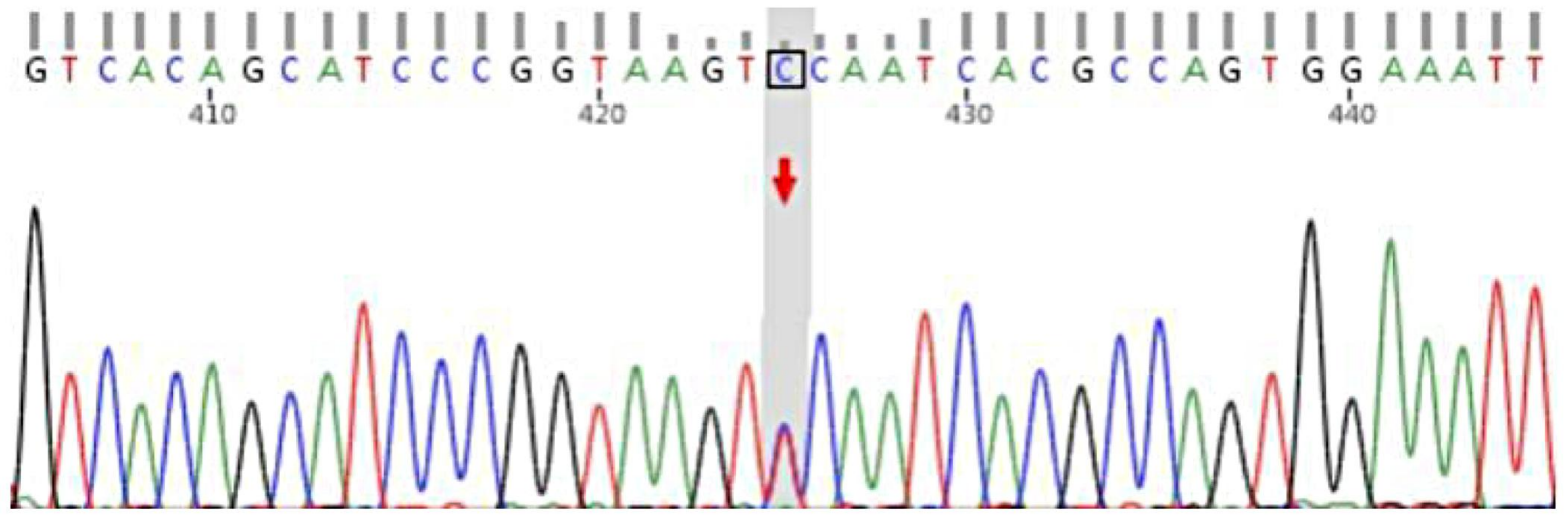			
Father, Reverse Sequencing Heterozygous		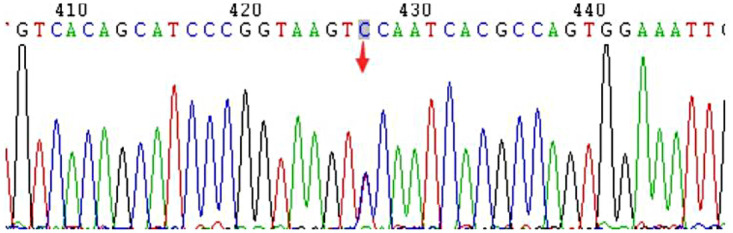			
Mother, Reverse Sequencing Wild-type		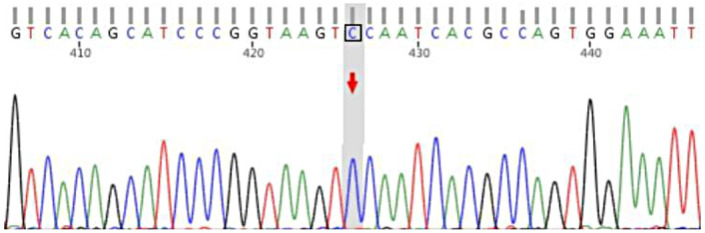			

### 3.5 Genetic annotation

The variant c.567dup (p.Ile190Tyrfs*13) carried by the proband is a frameshift variant arising from a non-triplet base duplication within the coding region of the *MMACHC* gene, which theoretically results in premature termination of protein translation. Multiple reports have documented pathogenic null variants downstream of this locus, indicating that the amino acid sequence downstream of this variant is critical to protein function. Pedigree sequencing of the proband confirmed maternal inheritance of this variant. In the large-scale population frequency database gnomAD, only 4 heterozygous carriers of this variant have been recorded, with no homozygous individuals identified. This variant has been detected in multiple patients with combined methylmalonic aciduria and homocystinuria in published literature (PMIDs: 21835369, 27383490, 30564975, etc.). Based on current available evidence, this variant is classified as a pathogenic variant.

The variant c.609G>A (p.Trp203*) carried by the proband is a nonsense variant within the coding region of the *MMACHC* gene, which theoretically causes premature termination of translation of the encoded protein. Multiple pathogenic null variants downstream of this variant have been reported, suggesting that the amino acid sequence downstream of this variant exerts a critical influence on protein function. Pedigree sequencing results of the proband demonstrated paternal inheritance of this variant. In the large-scale population frequency database gnomAD, 11 heterozygous carriers of this variant have been recorded, while no homozygous individuals have been reported. This variant has been identified in numerous patients with combined methylmalonic aciduria and homocystinuria in published literature (PMIDs: 20631720, 23954310, 28327205, etc.). Based on currently available evidence, this variant is classified as a pathogenic variant.

## 4 Discussion

MMA is an inherited metabolic disease caused by defects in methylmalonyl-CoA mutase or its coenzyme cobalamin (vitamin B12) metabolism, leading to abnormal accumulation of toxic metabolites such as methylmalonic acid and multi-system damage. cblC type is one of the most common subtypes, caused by *MMACHC* gene mutation, manifesting as MMA combined with homocysteinemia.

### 4.1 Clinical features and early identification

The patient in this case had neonatal-onset (within 20 days after birth) disease, with feeding symptoms and non-specific neurological abnormalities as the main manifestations: feeding difficulty, drowsiness, poor response, hypotonia, etc., without typical critical signs such as convulsions, severe metabolic acidosis and hyperammonemia. This atypical onset is not uncommon in the neonatal period, and is easily confused with neonatal infections (early-onset/late-onset sepsis), hypoxic-ischemic encephalopathy, improper feeding, congenital myopathy, Prader–Willi syndrome/Angelman syndrome (excluded by genetic testing), leading to delayed diagnosis. Literature reports that early-onset cblC type MMA can present with feeding difficulty, vomiting, drowsiness, dystonia, developmental delay, often accompanied by hematological abnormalities such as anemia and thrombocytopenia, and hydrocephalus in severe cases (5). Cranial MR of this patient showed hyperintensity in the globus pallidus on T1WI, which may be related to neurotoxicity caused by accumulation of metabolites, and is one of the common neurological imaging manifestations in MMA children (6). Persistent feeding difficulty and poor response are important clues suggesting potential inherited metabolic diseases. Clinicians, especially neonatologists and pediatricians, should remain vigilant for neonates and infants with unexplained feeding difficulty, poor mental response, abnormal muscle tone and growth retardation, and conduct corresponding examinations in a timely manner (7).

Consistent with the national multicenter cohort reported by Ling et al. (8), the compound heterozygous genotype c.609G>A/c.567dupT is an independent risk factor for neonatal-onset severe cblC-type MMA. The variant c.567dupT accounts for 4.9% of all pathogenic *MMACHC* alleles in Chinese newborn-screened patients, and patients harboring this variant combined with c.609G>A usually present with non-specific neonatal manifestations including feeding difficulty and hypotonia without severe metabolic decompensation, which coincides with the clinical features of our proband.

### 4.2 Diagnostic strategies and methods

The definitive diagnosis of cblC type MMA requires the combination of metabolic screening and genetic testing.

1. Metabolic screening is the cornerstone: Blood tandem mass spectrometry and urinary organic acid analysis are first-line methods for the diagnosis of MMA. Hyperhomocysteinemia is an important biochemical marker of cblC type (8). Typical biochemical characteristics are: elevated blood C3 and C3/C2 ratio, decreased or normal Met. Elevated serum homocysteine is the key index to distinguish isolated MMA from combined MMA (including cblC type), and significantly increased urinary methylmalonic acid is the key basis for the definitive diagnosis of MMA (9). The patient in this case conforms to the metabolic characteristics of MMA combined with homocysteinemia (10). Therefore, neonatal disease screening using tandem mass spectrometry can early detect children with elevated blood C3 and C3/C2 ratio, which is a key method to reduce the mortality and morbidity of this disease due to its low cost and reliable results.
2. Genetic testing is the gold standard: On the basis of biochemical screening suggesting cblC type, *MMACHC* gene testing can confirm the diagnosis and classification, and provide family genetic counseling and prenatal diagnosis. The *MMACHC* gene is located at 1p34.1 and contains 5 exons. In Chinese patients with cblC type, c.609G>A (p.Trp203*), c.658_660delAAG (p.Lys220del), c.80A>G (p.Gln27Arg), c.482G>A (p.Arg161Gln) are common hot-spot mutations (11, 12). Among them, the c.609G>A mutation has the highest detection frequency in Chinese patients, especially associated with early-onset and severe disease. The patient in this case carried two pathogenic variants, c.609G>A and c.567dupT, conforming to the compound heterozygous mutation pattern. Among them, c.609G>A was derived from the father, a confirmed hot-spot pathogenic mutation; c.567dupT was derived from the mother, also a reported pathogenic mutation. Both variants can lead to severe loss of MMACHC protein function, consistent with the neonatal-onset and relatively typical disease condition of the patient.

### 4.3 Treatment and prognosis

Once diagnosed, treatment should be started immediately. Most cblC type patients are responsive to vitamin B12. Acute phase requires correction of metabolic disorders (acidosis, hyperammonemia, etc.). Long-term treatment includes intramuscular injection of hydroxocobalamin (active vitamin B12), oral betaine (to reduce homocysteine), levocarnitine (to promote excretion of toxic metabolites), appropriate supplementation of folic acid and vitamin B6, and ensuring sufficient calorie intake. Strict restriction of natural protein is generally not required, but metabolic indicators need to be monitored regularly. Early diagnosis and timely standardized treatment can significantly improve the prognosis, and some children can achieve normal or near-normal neurodevelopment (13). If diagnosis and treatment are delayed, it can lead to irreversible neurological damage (mental and motor retardation, epilepsy, cerebral palsy, etc.), visual impairment, renal insufficiency, pulmonary hypertension and other serious complications, even death (14). The patient in this case was genetically diagnosed in the neonatal period, winning valuable time for early targeted treatment and improving long-term prognosis. Long-term follow-up is required for the patient, and the treatment plan should be adjusted timely.

### 4.4 Genetic counseling and prenatal diagnosis

This disease is inherited in an autosomal recessive manner. The parents of the patient are asymptomatic carriers. When they have another child, the offspring has a 25% chance of being affected, 50% chance of being a carrier, and 25% chance of being completely normal. Their first child was found to have hydrocephalus at 1 month after birth and died at the age of 9 years, which may also be this disease. After clarifying the mutations of the proband and parents through genetic testing, accurate genetic counseling can be provided for the family. During subsequent pregnancy, fetal DNA can be obtained through chorionic villus sampling or amniocentesis for prenatal genetic diagnosis to avoid the birth of children with the same disease.

## 5 Conclusion

Neonatal-onset cblC type methylmalonic acidemia combined with homocysteinemia has insidious and non-specific clinical manifestations, which are prone to missed diagnosis and misdiagnosis. Improving clinical vigilance is the key to early detection. For any neonate and infant with unexplained feeding difficulty, poor mental response, abnormal muscle tone and growth retardation, metabolic screening including blood tandem mass spectrometry, urinary organic acid analysis and serum homocysteine test should be conducted immediately. Genetic testing is the final basis for definitive diagnosis, classification, treatment guidance and family genetic counseling. The implementation of neonatal screening for inherited metabolic diseases, popularization of tandem mass spectrometry technology, and establishment of a multidisciplinary collaborative model for metabolic disease diagnosis and treatment and genetic counseling are the fundamental ways to achieve early diagnosis and treatment of this disease and improve the prognosis of patients.

## Data Availability

The original contributions presented in the study are included in the article/supplementary material, further inquiries can be directed to the corresponding author/s.
